# Antibiotic-Eluting Envelopes to Prevent Cardiac-Implantable Electronic Device Infection: Past, Present, and Future

**DOI:** 10.7759/cureus.13088

**Published:** 2021-02-02

**Authors:** Kun Xiang, John N Catanzaro, Claude Elayi, Zerelda Esquer Garrigos, Muhammad R Sohail

**Affiliations:** 1 Cardiology, University of Florida College of Medicine, Gainesville, USA; 2 Cardiology, University of Florida College of Medicine – Jacksonville, Jacksonville, USA; 3 Internal Medicine/Infectious Disease, Mayo Clinic College of Medicine, University of Mississippi Medical Center, Rochester, USA; 4 Cardiovascular Infectious Diseases, Baylor College of Medicine, Houston, USA

**Keywords:** cardiac implantable electronic device (cied), pocket infection, envelope, infection prevention and control, antibiotics pathogen

## Abstract

Objective: Cardiac-implantable electronic device (CIED) infections are associated with significant morbidity and mortality. In this review, we describe the risk factors and pathogenesis of CIED infections and review the rationale and the evidence for the use of antibiotic-eluting envelopes (ABEs) in patients at increased risk for CIED infections.

Findings: The majority of CIED infections are caused by staphylococci that involve generator pocket and occur due to contamination of the device or the pocket tissues at the time of implantation. Clinical trials have shown that extending the duration of post-operative systemic antibacterial therapy is not beneficial in reducing CIED infection rate. However, ABEs that reduce device migration after implantation and provide sustained local delivery of prophylactic antibiotics at the pocket site, may provide benefit in reducing infection. Currently, there are two types of commercially available CIED envelope devices in the United States. The first ABE device (TYRX™, Medtronic Inc., Monmouth Junction, NJ) is composed of a synthetic absorbable mesh envelope that elutes minocycline and rifampin and has been shown to reduce CIED pocket infections in a large multi-center randomized clinical trial. The second ABE device (CanGaroo-G™, Aziyo Biologics, Silver Spring, MD) is composed of decellularized extracellular matrix (ECM) and was originally designed to stabilize the device within the pocket, limiting risk for migration or erosion, and providing a substrate for tissue ingrowth in a preclinical study. This device has shown promising results in a preclinical study with local delivery of gentamicin. Compared with artificial materials, such as synthetic surgical mesh, biologic ECM has been shown to foster greater tissue integration and vascular ingrowth, a reduced inflammatory response, and more rapid clearance of bacteria.

Conclusions and Relevance: ABE devices provide sustained local delivery of antibiotics at the generator pocket site and appear beneficial in reducing CIED pocket infections. Given the continued increase in the use of CIED therapy and resultant infectious complications, innovative approaches to infection prevention are critical.

## Introduction and background

Infections are a devasting complication of cardiac-implantable electronic devices (CIED) therapy and are associated with increased morbidity, mortality, and cost. Moreover, reported rates of CIED infection are rising faster even when accounting for the continuous growth in CIED implantation [1]. This might be because of the CIED implantation in patients with multiple risk factors, such as older age and multiple comorbidities [1]. Prevention of CIED-related infection, therefore, is a key consideration in the design and implantation of CIEDs. Evidence-based prophylactic approaches include the use of preoperative antibiotics, thorough surgical skin disinfection, and the implantation of CIED with antibiotic-eluting envelopes [2]. Emerging evidence supports the efficacy of these antibiotic-eluting envelopes that provide high local antibiotic concentrations in the surgical pocket, with minimal systemic exposure [3]. Currently, there are two commercially available CIED envelope devices in the United States. This article reviews the evidence describing the rationale for these antibiotic-eluting envelopes in patients undergoing CIED implantation.

## Review

CIED infections: Incidence, costs, and risk factors

In the absence of comprehensive and mandatory registries, the estimates of the incidence of CIED infections in North America mostly rely on observational studies and vary based on study type, CIED type, and follow-up duration. A recent analysis of a claims database from 2016 reported a cumulative incidence of CIED infection within one-year post-implant of 1.18% for initial implantation and 2.37% for device replacement [4]. Other studies have reported an incidence of CIED-related infection as high as 7% following device reintervention [5]. Although studies report a wide range of estimated costs of managing CIED infection, depending on the patient population and the need for device removal and/or hospitalization, the costs are consistently high [6].

Risk factors for CIED infection include both patient and procedural factors and are summarized in Table 1 [6-8].

**Table 1:** Risk factors for CIED infection CIED: cardiac-implantable electronic device.

Patient factors	Procedural factors
Age, previous valvular surgery, device reimplantation, renal failure or dialysis, heart failure, chronic lung disease, cerebrovascular disease, recent fever (≤24 hours), development of hematoma, diabetes, corticosteroid use, oral anticoagulation	Presence of temporary wire, need for re-intervention, use of surgical drains, multiple procedures, type of CIED, duration of the procedure, presence of preexisting transvenous leads (abandoned), length of the procedure

Microbiology and pathogenesis of CIED-related infections

**Figure 1:**
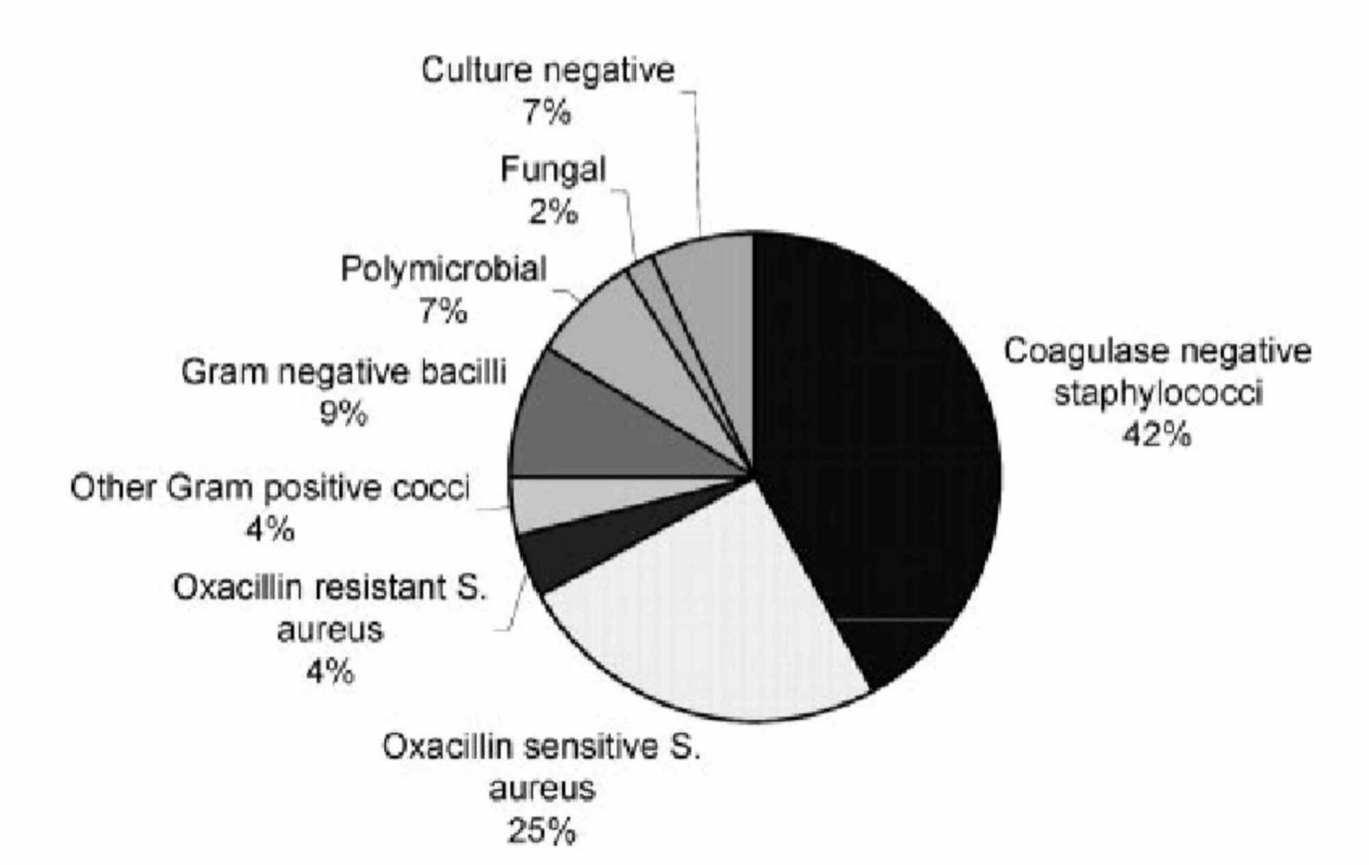
Common microbiology and pathogenesis of CIED-related infections CIED: cardiac-implantable electronic device.

Coagulase-negative Staphylococci (primarily *Staphylococcus epidermidis*) and *S. aureus* account for >70% of all CIED infections (Figure 1) [9]. Less common pathogens include Gram-negative cocci and fungi. The prevalence of antibiotic-resistant Staphylococcus spp. (particularly *S. aureus*) differs based on the study and geographic location of the study population and must be considered when managing CIED infections [2]. Clinical presentation ranges from local infection of the surgical pocket to bacteremia and endocarditis [2]. Majority of CIED-related infections due to contamination of the pocket by skin flora during the initial implantation or subsequent revision procedures. Pocket infection can also occur following erosion of the device or leads through the skin. Infection of the CIED pocket can then propagate along the leads into the intravascular system, resulting in bloodstream infection and valvular endocarditis. Occasionally, patients may develop hematogenous seeding of the electrode or pocket from a distant source [2].

The initial attachment of microorganisms to the device is mediated by the characteristics of the organism and the device surface. Microbes may also adhere to extracellular matrix (ECM) proteins produced by the host that coat the device surface in the pocket; this mechanism is particularly relevant to new device technologies, such as CIED envelopes. As additional bacteria attach to the bacteria already adhered to the device surface, a biofilm of bacterial layers and intercellular adhesins develops, which makes the bacteria more resistant to antibiotics and immune defenses, specifically by inhibiting penetration to the local area [2].

Prevention of CIED-related Infections

Standard Prophylaxis (Excluding Antibiotic-Eluting Device Envelopes)

Prophylaxis is the preferred strategy to reduce morbidity and mortality associated with CIED infection [2]. Evidence-based approaches to reduce infection risk include preoperative administration of antibiotics and thorough skin antisepsis [2]. Parenteral antibiotics administered prior to implantation procedure have been shown to significantly reduce the incidence of device-related infections [2,10]. In one double-blind trial, 649 patients were randomized to IV cefazolin or placebo, both administered immediately before the procedure [10]. The trial was terminated early due to clear evidence of benefit; infections occurred in 0.63% of the cefazolin group and 3.28% of the placebo group (relative risk 0.19; P=0.016). Based on this landmark trial, the American Heart Association recommends the administration of parenteral antibiotics within an hour of CIED procedures [2].

Skin antisepsis is another standard preoperative technique for reducing CIED infection risk [2]. Some evidence suggests benefits to the use of chlorhexidine-alcohol over povidone-iodine, mostly based on a single randomized study of patients undergoing clean-contaminated surgeries, mostly abdominal and thoracic (not CIED-related). This study reported a significantly lower infection rate with chlorhexidine-alcohol compared to povidone-iodine (9.5% vs. 16.1%; P=0.004) [11]. Other common prophylactic techniques include optimization of the patient’s clinical status, a chlorhexidine scrub (Hibiclens) prior to the procedure, eradication of nasal bacteria methicillin-resistant *S. aureus* (MRSA), and irrigation of the surgical pocket with antibiotics. It is worth noting that evidence does not definitively support their efficacy for the prevention of CIED infections [12]. Similarly, there is currently no evidence to support the use of postoperative antibiotics [8]. However, despite the minimal evidence, these strategies are common in clinical practice. In the large cohort of 6983 patients in the recently reported WARP-IT study, 74.5% received pocket irrigation with antimicrobial solutions, and 29.6% received post-procedure antibiotics [3].

Antibiotic-Eluting CIED Envelopes

Antimicrobial-impregnated CIED envelopes have been developed to hold devices securely in place to provide a stable environment and to reduce infection risk [13]. These devices deliver a prolonged, high concentration of antibiotics effective against common CIED pathogens within the CIED pocket. Because most CIED contaminations are believed to occur at the time of implantation, these envelopes should provide an effective method of CIED infection prophylaxis. Evidence from other surgical procedures supports the efficacy of local antibiotic delivery to prevent wound infections [14-16]. For example, the use of gentamicin-impregnated collagen implants significantly reduces the risk for sternal wound infections in high-risk patients undergoing cardiac surgery [15-17]. Studies of this collagen-based technology identified high gentamicin concentrations at the surgical site and very low serum drug levels, suggesting that antibiotic is concentrated at the site of potential infection while minimizing systemic exposure and the risk for adverse events or the development of bacterial resistance [16-19].

**Figure 2:**
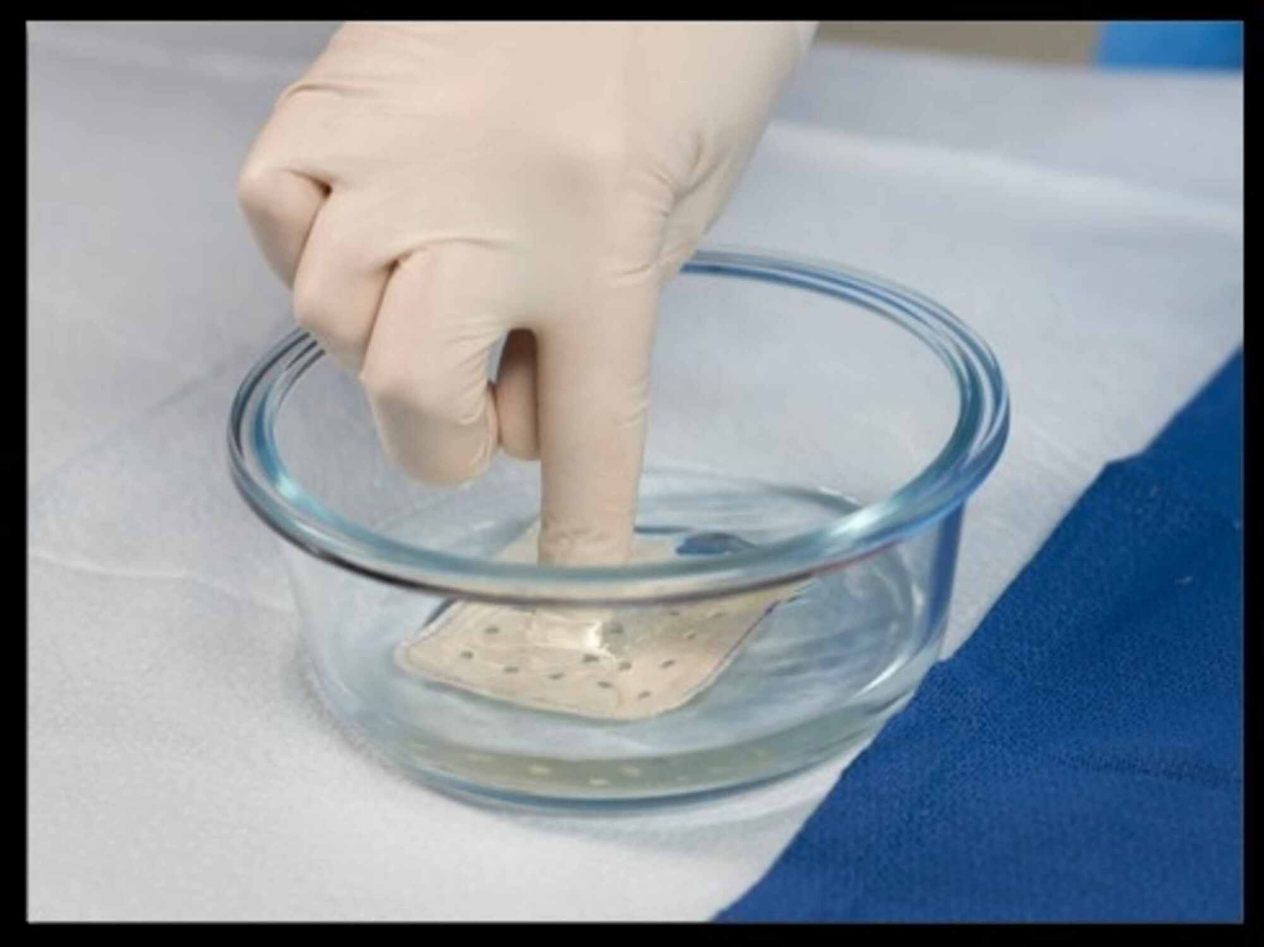
ECM is hydrated in a gentamicin solution prior to implantation ECM: extracellular matrix.

Two CIED envelope devices are currently available for use in the US. Both are absorbable. One is made from absorbable multifilament block copolymer comprised of glycolide, caprolactone, and trimethylene carbonate mesh that is coated with an absorbable polyacrylate polymer containing minocycline and rifampin. The other envelope is made from decellularized, non-crosslinked ECM, which is derived from porcine intestinal submucosa; this device is hydrated in a gentamicin solution prior to implantation (Figure 2). These envelopes are designed to stabilize the CIED within the pocket, limiting the risk for migration or erosion. The envelopes also provide a substrate for tissue ingrowth and allow for the local delivery of antibiotics.

Synthetic Mesh Envelope

The CIED envelope made of synthetic mesh (TYRX™; Medtronic, Inc., Monmouth Junction, NJ) elutes minocycline and rifampin for a minimum of seven days. An earlier version of the device (AEGIS) consisted of non-absorbable polypropylene but was associated with significant pocket fibrosis, making removal of a generator and/or leads difficult. A newer version was developed that is fully absorbed within approximately nine weeks after implantation. Initial retrospective studies suggested potential benefits to this device, and these findings were further supported by the prospective CITADEL and CENTURION studies [20-22]. Combined analysis of these studies identified a significantly lower CIED-related infection rate among prospectively enrolled patients treated with the synthetic mesh envelope, compared to historical controls (0.4% vs. 2.2%; P=0.0023) [22]. A recent meta-analysis of five cohort studies reported a pooled odds ratio of 0.31 (0.17, 0.58, 95% CI, P=0.0002) for CIED infection with versus without antibacterial envelopes for CIED implantation [23].

Importantly, the efficacy of the synthetic envelope was recently reported in a prospective multicenter randomized clinical trial (WRAP-IT 2019) [3]. This trial enrolled 6983 patients undergoing CIED implantation, replacement, or revision to receive the synthetic mesh envelope or not (control). Overall, 25 patients in the envelope group and 42 patients in the control group met the primary endpoint (infection resulting in device extraction or revision, long-term antibiotic therapy with infection recurrence, or death) within 12 months of the procedure (12-month estimated event rate 0.7% vs. 1.2%, respectively; P=0.04). In addition, long-term follow (36 months) of WRAP-IT study reported major CIED-related infections occurred in 32 envelope patients and 51 control patients (Kaplan-Meier [KM] estimate 1.3% vs. 1.9%; hazard ratio [HR] 0.64; 95% confidence interval [CI] 0.41-0.99; P=0.046). Any CIED-related infection occurred in 57 envelope patients and 84 control patients (KM estimate 2.1% vs 2.8%; HR 0.69; 95% CI 0.49-0.97; P=0.030). System- or procedure-related complications occurred in 235 envelope patients and 252 control patients (KM estimate 8.0% vs. 8.2%; HR 0.95; 95% CI 0.79-1.13; P<0.001 for noninferiority) [24].

Although the results of this well-designed study (WRAP-IT) achieved statistical significance (i.e., P<0.05), the findings were not as robust as anticipated due to lower than anticipated overall infection rate. Moreover, the number-needed-to-treat (NNT) for this study to prevent one CIED infection was 200 patients, an elevated number compared to other preventive therapies. Therefore, further refinements are needed to optimize the clinical benefits and costs of these novel envelopes.

ECM Envelope

Another CIED envelope that uses different materials and antibiotics is currently available. The CanGaroo-G™ envelope (Aziyo Biologics, Silver Spring, MD) is made with a biologic material that utilizes ECM, rather than synthetic mesh, to create a bioscaffold for tissue integration and neovascularization (Figure 2). This ECM envelope is made of a four-layer biomaterial derived from decellularized, non-crosslinked porcine intestinal submucosa. Prior to implantation, the ECM envelope is hydrated in a gentamicin solution (20 mL of 40 mg/mL solution) for two minutes. The ECM envelope can be used for transvenous pacemakers as well as subcutaneous implantable cardioverter defibrillators (SICD). Xiang and Levine described the first published clinical procedural use of the SICD envelope to anchor the SICD to the thoracic wall [25]. Preclinical studies suggest that the ECM envelope delivers high concentrations of gentamicin to the surrounding tissues, with minimal systemic exposure, providing excellent efficacy against Staphylococcus spp. and other CIED pathogens [26]. Importantly, the release of gentamicin from the ECM envelope peaks early (within one hour), followed by a gradual, sustained release over one week. This pharmacokinetic profile matches the presumed mechanism of CIED infection, which most often involves contamination of the pocket by skin flora at the time of implantation. In the recently published preclinical animal infection model, no bacteria were recovered from any culture after 12 hours of exposure to the gentamicin containing ECM envelope. Serum gentamicin levels dropped below the limit of quantification at 15 hours after implant [27]. Gentamicin concentration in the ECM envelope remained relatively stable for up to seven days [27]. These findings suggest that gentamicin containing ECM envelope is effective in reducing bacterial burden in the implant pocket.

Synthetic vs. biologic materials

Compared with artificial materials, such as synthetic surgical mesh, biologic ECM has been shown to foster greater tissue integration and vascular ingrowth, a reduced inflammatory response, and more rapid clearance of bacteria [28-33]. Numerous studies have demonstrated that ECM-based materials, particularly non-crosslinked materials, promote site-specific functional tissue remodeling [28,29]. Non-crosslinked biologic materials have also been shown to minimize foreign body response, inflammation, and fibrosis and foster angiogenesis and cell differentiation as part of the remodeling process [30,31]. As a result of reduced inflammation and enhanced tissue ingrowth and angiogenesis, these biologic materials can promote the elimination of bacteria and thereby reduce infection risk [32]. Moreover, some studies suggest that ECM bioscaffolds have inherent bactericidal activity [33]. Certain antibacterial factors are active within the intact ECM, whereas others are released during modification of the matrix after implantation. It has been suggested that components of intact ECM promote a transformation in macrophage phenotype from predominantly pro-inflammatory (called M1) to anti-inflammatory (M2). M1-type macrophages mediate tissue damage and promote an inflammatory response [34]. Infiltration of M2-type macrophages has been shown in the early stages of tissue repair, and depletion of M2-type macrophages has been shown to inhibit the formation of vascularized granulation and scar tissues [35]. Repair materials with different characteristics stimulate differing macrophage responses, and a correlation has been shown between early macrophage response and tissue remodeling outcomes [36]. A greater proportion of M2 macrophages has been associated with positive remodeling outcomes, as occurs with non-cross-linked ECM. In contrast, synthetic materials and highly cross-linked biologic materials typically trigger a more robust foreign body response, including chronic inflammation and fibrous encapsulation of the material, rather than integration into host tissues [28,31].^ ^The inflammatory response to synthetic and cross-linked materials is characterized by a predominance of M1 phenotype, which facilitates the formation of scar tissue and encapsulation of the material [35,37]. Based on this evidence, biologic repair materials are generally preferred over synthetic materials in contaminated surgical fields [38].

Envelope-delivered antibiotics

Studies of multiple surgical procedures have reported reduced infection rates with the local application of antibiotics. Based on this evidence, the microbiology of CIED infections, and bactericidal data, current CIED envelope devices utilize either rifampin/minocycline or gentamicin for infection prophylaxis.

Rifampin and Minocycline

Rifampin inhibits bacterial DNA-dependent RNA synthesis, whereas minocycline inhibits bacterial protein synthesis. Both agents are active against Staphylococcus spp. [8]. The reported MIC of rifampin for *S. aureus* and coagulase-negative Staphylococci isolated from CIED infections is ~0.5 to 2 mg/L [39]. In vitro studies have demonstrated rifampin has activity against *S. epidermidis* biofilms, especially in combination with certain other agents, whereas many other tested antibiotics were ineffective [40]. The addition of rifampin to minocycline has been shown to potentiate its in vitro antibacterial effect, including on resistant strains such as MRSA [41]. The combination of both drugs also reduces resistance to rifampin [42].

The combination of rifampin/minocycline has been used extensively in antibiotic-impregnated catheters. Studies report significant reductions in the risk for infection with rifampin/minocycline-impregnated catheters compared to standard catheters in neurosurgical procedures [43], chemotherapy administration [44], and other central venous catheter applications [45]. Studies of the synthetic mesh CIED envelope, which elutes a combination of rifampin and minocycline, have demonstrated a reduction in bacterial contamination in rabbit models, including the elimination of Staphylococcus spp., *Escherichia coli*, and other bacterial species [46]. The aforementioned WRAP-IT study demonstrated significant (P<0.05) reductions in CIED-related infections with the rifampin/minocycline synthetic envelope, compared to no envelope [3].

Gentamicin

Gentamicin is an aminoglycoside that inhibits bacterial protein synthesis and has a broad-spectrum bactericidal activity that includes Staphylococcus spp. and aerobic Gram-negative organisms. Susceptibility studies demonstrate >3-log reductions in bacterial colonization (i.e., >99.9% reductions), both in vitro and in vivo including the bacteria most commonly isolated from CIED infections. The minimum inhibitory concentration (MIC) for gentamicin for common CIED pathogens (e.g., Staphylococcus spp.) has been reported to be ~0.5 to 4 mg/L [39,47].

Gentamicin has demonstrated efficacy in vitro against Staphylococcal biofilms; in one study, only daptomycin had similar efficacy among single-agent antibiotics [48]. Similarly, an in vitro pharmacodynamic study demonstrated 3-log reductions in *S. aureus* inoculates across a sixfold range of gentamicin concentrations [49].

Some clinical data and animal studies also suggest the antimicrobial efficacy of gentamicin for the prevention of CIED-related infections [27]. Local use of gentamicin has been shown to reduce infection rates in patients undergoing CIED implantation [24,50].

The main limitation of the systemic use of gentamicin is its association with risk for nephrotoxicity and ototoxicity. Consequently, systemic administration of aminoglycosides has been used sparingly for several decades, thereby limiting the development of resistance to this class of antibiotics. Conversely, as described above, local administration of gentamicin has demonstrated efficacy in multiple surgical procedures, without the risks of systemic exposure [15-17,23,50].

The effects of gentamicin are concentration-dependent, requiring a high concentration in the surgical site to produce high bactericidal activity, even against organisms with low susceptibility or resistance to this agent. When administered into the surgical site, gentamicin concentrations in local tissues can exceed 300 mg/L, whereas systemic exposure is limited to ~1-2 mg/L [18]. In one study of sternal wound infection, treatment using a collagen implant loaded with gentamicin was effective, even when antibiotic-resistant *S. epidermidis* was present, presumably due to the high local concentrations of gentamicin [18]. Therefore, local administration of gentamicin using an ECM envelope provides high early tissue concentrations of antibiotics, with minimal systemic exposure.

Currently, the efficacy of gentamicin delivering ECM envelope has been reported in preclinical animal infection models and clinical case report [25,27]. A prospective randomized clinical study is needed to prove the safety and efficacy of CIED infection reduction. It is worth mentioning that a similar preclinical animal model was used earlier to investigate the safety and efficacy of minocycline/rifampin synthetic envelope [46]. The results of such animal experiments were later confirmed in a prospective randomized clinical trial [3].

## Conclusions

Minimizing risks of adverse outcomes associated with CIED implantation, especially infection, is of paramount importance to prevent serious complications that warrant device removal. New CIED envelope technologies demonstrate promise to reduce generator pocket infection. Preclinical and clinical data support the efficacy of local antibiotic delivery through synthetic or ECM-based CIED envelopes eluting rifampin/minocycline and gentamycin, respectively. Moreover, the ECM envelope typically stimulates tissue remodeling and angiogenesis, thereby minimizing inflammation and promoting bacterial clearance. Large, prospective studies are needed to help refining indications and judicious use of the ECM envelope to prevent CIED infection.
